# Distinct metabolic architectures differentiate HLB-tolerant lemon and susceptible sweet orange

**DOI:** 10.3389/fpls.2026.1905341

**Published:** 2026-08-11

**Authors:** Paul Gomes, Baoping Cheng, Benjamin Laffitte, Muhammad Saqib Bilal, Muhammad Naveed Aslam, Qian Chen, Taiyun Wei, Hongwei Zhao

**Affiliations:** 1 State Key Laboratory of Agricultural and Forestry Biosecurity, College of Plant Protection, Nanjing Agricultural University, Nanjing, China; 2 Plant Protection Research Institute, Guangdong Academy of Agricultural Sciences, Key Laboratory of Green Prevention and Control on Fruits and Vegetables in South China Ministry of Agriculture and Rural Affairs, Guangdong Provincial Key Laboratory of High Technology for Plant Protection, Guangzhou, China; 3 State Key Laboratory of Geohazard Prevention and Geoenvironment Protection, Chengdu University of Technology, Chengdu, China; 4 College of Ecology and Environment, Chengdu University of Technology, Chengdu, China; 5 Key Laboratory of Ecological Safety and Sustainable Development in Arid Lands, Xinjiang Institute of Ecology and Geography, Chinese Academy of Sciences, Urumqi, China; 6 Department of Plant Pathology, Faculty of Agriculture and Environment, The Islamia University of Bahawalpur, Bahawalpur, Pakistan; 7 State Key Laboratory of Agricultural and Forestry Biosecurity, College of Plant Protection, Fujian Agriculture and Forestry University, Fuzhou, China

**Keywords:** bootstrap-enhanced LASSO, citrus, disease tolerance, Huanglongbing, metabolomics, phenylpropanoids

## Abstract

Huanglongbing (HLB), caused by *Candidatus* Liberibacter asiaticus (*C*Las), is a devastating citrus disease that severely threatens global citrus production, yet the metabolic mechanisms underlying citrus tolerance to HLB remain elusive. Citrus genotypes exhibit differential tolerance to HLB, with lemon (*Citrus limon*) showing greater resilience compared to susceptible sweet orange (*Citrus sinensis*). In this study, we integrated untargeted liquid chromatography-high resolution mass spectrometry metabolomics with a bootstrap-enhanced LASSO approach to characterize the metabolic responses of HLB-tolerant *C. limon* and susceptible *C. sinensis* to *C*Las infection. A total of 1,294 metabolites were identified. Bootstrap-enhanced LASSO identified stable discriminant metabolites (DMs) across four comparative frameworks, with phenylpropanoids (flavonols, furanocoumarins, and hydroxycinnamic acids) and limonoids being the most prominent DM classes. Lemon exhibited constitutively higher levels of kaempferol/quercetin-derived flavonols, linear furanocoumarins, monolignol glycosides, and limonoids compared to orange. While *C*Las infection induced quantitative changes in specific metabolic branches, the overall metabolic organization remained primarily determined by genotype. In the two cultivars examined, these results suggest that pre-existing metabolic configurations distinguish the tolerant lemon from the susceptible sweet orange and are associated with their contrasting HLB responses. This study provides robust metabolic signatures and candidate pathways for improving HLB tolerance through citrus breeding programs.

## 1 Introduction

Huanglongbing (HLB), also known as citrus greening, is a bacterial disease affecting citrus worldwide. It is caused by the fastidious, phloem-limited, Gram-negative bacteria of the genus *Candidatus* Liberibacter, primarily *Ca*. L. asiaticus (*C*Las), as well as *Ca.* L. americanus and *Ca*. L. africanus. It is transmitted by insect vectors such as the Asian citrus psyllid (*Diaphorina citri)* while feeding on phloem sap (Bové, 2006). HLB is considered one of the most devastating citrus diseases, severely impacting fruit production and quality, and threatening the world’s citrus industry. Since its detection in Florida in 2005, HLB has led to a dramatic drop in citrus production of more than 60% in two decades, resulting in billions of dollars of losses (Singerman and Rogers, 2020). Similar impacts have been observed in China, where HLB has been present for more than a century and remains a major constraint on citrus production, particularly in southern provinces such as Guangdong, Guangxi and Fujian (Zheng et al., 2018). Typical symptoms of infected trees include mottled leaves, the production of small, deformed fruits that remain partially green and bitter-tasting, and a gradual decline of the tree over the years (Bové, 2006).

Commercial citrus production relies largely on HLB-susceptible cultivars, including most sweet orange (*Citrus sinensis*) genotypes (Bové, 2006). At the cellular level, *C*Las infection induces major physiological disruptions. Callose deposition occludes sieve plates and impairs symplastic transport (Koh et al., 2012). As the disease progresses, phloem cells eventually collapse, disrupting source-sink dynamics, thereby leading to a gradual decline of the host (Brodersen et al., 2014). In addition, *C*Las infection triggers a deregulated accumulation of reactive oxygen species (ROS) in phloem tissues, inducing oxidative stress that ultimately leads to the death of companion cells and sieve elements (Ma et al., 2022). To date, no fully resistant commercial citrus variety has been identified. Some varieties, such as lemon (*Citrus limon*), exhibit greater tolerance, characterized by milder symptoms and the ability to maintain relatively normal growth for an extended period of time (Miles et al., 2017). Most commercially important citrus varieties are hybrids derived from three ancestral taxa: citron (*C. medica*), pomelo (*C. maxima*), and mandarin (*C. reticulata*). Phylogenetic studies show that varieties whose pedigrees include *C. medica*, such as *C. limon*, exhibit greater resilience to HLB (Miles et al., 2017). Tolerant and susceptible citrus show different biochemical responses to *C*Las infection. Previous studies have highlighted differences in processes associated with antimicrobial defense, oxidative stress management, and reinforcement of the cell wall (Bilal et al., 2024; Gao et al., 2023; Hijaz et al., 2020; Cheng et al., 2023). Together, these mechanisms could contribute to limiting *C*Las colonization and maintaining vascular functionality. However, the way these different layers of defense are coordinated at the biochemical level in more resilient genotypes remains poorly understood. Further exploration of these molecular differences may clarify the processes involved in HLB tolerance.

Plant responses to biotic stresses often rely on the reprogramming of their secondary metabolism. In the case of HLB, the tolerance observed in lemon suggests the existence of specific defense mechanisms capable of reducing the impact of *C*Las, mitigating cellular damage caused by infection, or reinforcing host tissues. To explore such complex processes, untargeted metabolomics offers a powerful approach, as it provides a broad and unbiased metabolic profile of the plant, including both well-known and unknown metabolites. Liquid chromatography coupled with high-resolution mass spectrometry (LC-MS) enables the identification of complex metabolic signatures (Cajka and Fiehn, 2016). Previous citrus metabolomics studies have shown that HLB infection is associated with broad metabolic remodeling. Early metabolomic comparisons distinguished tolerant citrus relatives or rootstocks from susceptible sweet orange and grapefruit, suggesting that basal metabolic composition may contribute to differential HLB responses, although these studies mainly emphasized global metabolic separation or primary-metabolite variation rather than specific defense-related secondary metabolic branches (Cevallos-Cevallos et al., 2012; Albrecht et al., 2016). Other analyses in infected sweet orange revealed major changes in carboxylic acids, amino acids, fatty acids, pigments, and other metabolites, providing important insight into HLB-associated metabolic disruption but focusing largely on susceptible backgrounds or disease-associated responses rather than tolerance-specific organization (Killiny and Nehela, 2017). In contrast, comparative studies involving tolerant genotypes suggested that higher phenolic and flavonoid contents, antioxidant capacity, and growth- or phloem-regeneration-associated processes may contribute to HLB tolerance (Hijaz et al., 2020; Suh et al., 2021). More recently, temporal metabolomics showed that CLas infection can trigger early metabolic responses, including flavonoid-related changes, but such short-term responses do not fully clarify whether tolerant genotypes are characterized by inducible defenses, pre-existing metabolic configurations, or both (Li et al., 2024). Plant defense involves both pre-existing biochemical features and defenses activated upon stress. Untargeted metabolomics represents a powerful approach to capture both types of variation, providing a comprehensive view of the biochemical processes underlying HLB tolerance (Cajka and Fiehn, 2016; Suh et al., 2021).

Metabolomics datasets are typically high-dimensional, making the identification of reliable discriminant features statistically challenging. Although multivariate methods are widely used for class discrimination, variable selection may be unstable when sample size is limited and metabolites are highly correlated. To address this issue, we combined LASSO-based feature selection with bootstrap resampling to prioritize metabolites consistently selected across repeated models (Bach, 2008; Meinshausen and Bühlmann, 2010).

In this study, we aimed to characterize and compare the metabolic responses of the HLB-tolerant *C. limon* and the susceptible *C. sinensis* to *C*Las infection and discover biochemical patterns associated with HLB tolerance. By integrating untargeted high-resolution LC-MS metabolomics with a robust feature selection method adapted to high-dimensional datasets, we identified pathways and molecular signatures discriminating between the two genotypes. We hypothesized that the milder symptoms observed in lemon result from a combination of constitutive metabolic traits and distinct reprogramming upon infection. The identification of robust and stable metabolic signatures provides a list of compounds and specific metabolic branches that may support future citrus breeding programs.

## 2 Materials and methods

### 2.1 Plant material and *C*Las inoculation

One-year-old plants of *C. limon* ‘Xiangshui’ and *C. sinensis* ‘Newhall’ were used in this study. Plants were inoculated with *C*Las by grafting buds collected from *C*Las-positive donor trees. Mock-inoculated plants were grafted in the same way using buds from *C*Las-free donor trees. Each plant was randomly assigned to the *C*Las-inoculated or mock-inoculated groups before grafting. Four buds were grafted onto each plant. Prior to inoculation, *C*Las-positive donor material was screened by real-time quantitative PCR (qPCR), and donor buds with comparable *C*Las titers were selected for both lemon and sweet orange in order to minimize differences in initial inoculum pressure. Seedlings were obtained from Qingyuan National Citrus Seedling Breeding Base, Guangdong, China, and maintained in a greenhouse under controlled temperature conditions of 25–28 °C under a 16 h light/8 h dark photoperiod. Leaf samples were collected 24 weeks after graft inoculation. For each citrus variety and treatment condition, six independent biological replicates were collected, with each replicate corresponding to a single tree. For each tree, the canopy was separated into four orientations: south, east, north, and west. Two fully expanded mature symptomatic leaves were sampled from each orientation, and comparable mature leaves in mock-inoculated plants, giving a total of eight leaves per tree. Leaf tissues from each tree were pooled and ground together in liquid nitrogen. The same pooled material was then divided into aliquots, with one aliquot used for qPCR-based verification of infection status and quantification of *C*Las and another used for LC–MS-based metabolomic analysis. Thus, the infection status of each biological replicate was determined using material derived from the same pooled leaf sample used for metabolomic profiling. The resulting aliquots were immediately frozen in liquid nitrogen and stored at −80 °C until DNA or metabolite extraction.

### 2.2 *C*Las detection

For CLas quantification, 200 mg of frozen pooled leaf powder from each biological replicate was used for DNA extraction. The tissue was ground to a fine powder in liquid nitrogen using a mortar and pestle. Genomic DNA was extracted using a Plant DNeasy kit following the manufacturer’s protocol (QIAGEN, Hilden, Germany). *C*Las detection and quantification were carried out by qPCR using primers and probes targeting the *C*Las 16S rDNA gene. A plasmid containing a fragment of the *C*Las 16S rDNA sequence was used to generate the standard curve. Plasmid DNA was extracted according to the manufacturer’s instructions (TaKaRa, Japan), and 10-fold serial dilutions were prepared to obtain standards ranging from 10^9^ to 10¹ plasmid copies per reaction. Cycle threshold (Ct) values obtained from the dilution series were used to establish a linear relationship between Ct and log_10_-transformed 16S rDNA copy number. The resulting standard curve equation was: log_10_(copy number) = −0.286 × Ct + 11.924. For each sample, *C*Las 16S rDNA copy number was calculated from the Ct value using the following equation: T = 100 ×10^(-0.286 × Ct + 11.924)^, where T represents the *C*Las titer, expressed as 16S rDNA copy number per gram of fresh weight (FW), and Ct corresponds to the qPCR cycle threshold value. Each 20 μL qPCR reaction contained 250 nmol/L primers, 150 nmol/L probe, 1 μL DNA template, and qPCR master mix according to the manufacturer’s instructions. Amplification was performed on a LightCycler^®^ 480 II system (Roche, Switzerland) using the following program: initial denaturation at 95 °C for 20 s, followed by 40 cycles of 95 °C for 5 s and 58 °C for 40 s.

### 2.3 Metabolite extraction

Approximately 50 mg of frozen leaf tissue was weighed into 1.5 mL tubes and extracted with 800 µL of precooled methanol:H_2_O (7:3, v/v) and 20 µL of internal standard (IS1). Homogenization was conducted using a bead mill at 50 Hz for 10 min, followed by water bath ultrasonication at 4 °C for 30 min. Extracts were incubated at -20 °C for 1 hour and centrifuged at 14,000 rpm for 15 min at 4 °C. Supernatants (600 μL) were filtered through 0.22 μm membranes. An aliquot (20 μL) from each sample was pooled to generate a quality control (QC) sample to evaluate the repeatability and stability of the analysis. Filtered samples and QC samples were transferred to 1.5 mL vials for LC-MS analysis.

### 2.4 LC-MS analysis

Metabolomic profiling was performed using a Waters UPLC I-Class Plus system combined with a Q-Exactive high-resolution mass spectrometer (Thermo Fisher Scientific, USA). Chromatographic separation was performed on a Hypersil GOLD aQ column (1.9 μm, 2.1 x 100 mm, Thermo Fisher Scientific, USA). The mobile phase A consisted of 0.1% formic acid in water and mobile phase B consisted of 0.1% formic acid in acetonitrile. The gradient conditions were as follows: 5% B (0.0-2.0 min), 5-95% B (2.0-22.0 min), 95% B (22.0-27.0 min) and column washing with 95% B (27.1–30 min). Flow rate was 0.3 mL/min and the injection volume was 5 μL. To avoid potential bias from instrument drift, all samples were analyzed in a randomized order via LC-MS. Mass spectra were acquired in both positive and negative ion modes. The scan range was 125–1500 m/z (positive) and 100–1500 m/z (negative) at a resolution of 70,000. The automatic gain control (AGC) target for MS acquisitions was set to 1 x 10^6^ with a maximum ion injection time of 100 ms. Data-dependent acquisition (DDA) mode was used for MS/MS analysis. The top three most intense precursors were selected for subsequent MS/MS fragmentation at a resolution of 30,000, with an AGC target of 2 x 10^5^ and a maximum ion injection time of 50 ms. The stepped normalized collision energy was set to 20, 40 and 60 eV. ESI source parameters were set as follows: sheath gas flow rate 40 (arbitrary units), auxiliary gas flow rate 10, spray voltage 3.80 kV (positive mode) and 3.20 kV (negative mode), capillary temperature 320 °C, and auxiliary gas heater temperature 350 °C.

### 2.5 Peak extraction and metabolite identification

Raw data were processed using Compound Discoverer 3.3 software (Thermo Fisher Scientific, USA) for peak detection, alignment, and deconvolution. The following parameters were applied: parent ion mass tolerance < 5 ppm, fragment ion mass tolerance < 10 ppm, and retention time alignment tolerance< 0.2 min. Metabolite annotation was performed by matching MS/MS fragmentation patterns, accurate mass, and retention time against the BGI metabolome database, mzCloud and ChemSpider databases. Metabolite annotation confidence was assigned using the five-level scheme implemented in the BGI untargeted metabolomics pipeline, based on accurate MS1 mass, MS/MS fragmentation, retention time, database matching, and the availability of reference-standard information. Level 1 corresponded to annotations supported by matching against the BMDB reference-standard library and associated laboratory data; Level 2 to structural annotations supported by MS/MS spectral-library matching; Level 3 to tentative annotations supported by partial MS/MS spectral or structural matching and requiring further verification; Level 4 to database matches based only on accurate MS1 mass; and Level 5 to unidentified features without database matches. Annotation confidence levels are reported in **Supplementary Table 2**. The data matrix containing metabolite peak intensities and identification information was exported for data preprocessing.

### 2.6 Data preprocessing and quality control

Data preprocessing was performed using the MetX platform. Probabilistic quotient normalization was applied to normalize peak intensities. Quality control-based robust LOESS signal correction was applied to correct batch effects. Compounds with a coefficient of variation of relative peak area > 30% across QC samples were discarded. After normalization and quality filtering, a total of 1,294 metabolites were retained. Because of the high number of features relative to the number of biological samples, two unsupervised filtering steps were applied before LASSO analysis. First, metabolites were ranked according to their overall normalized LC–MS intensity across all samples, and the top 50% were retained. Intensity-based filtering was used to reduce the lowest-signal portion of the dataset, as low-intensity LC–MS features may be closer to the analytical background or detection limit and more prone to incomplete detection (Schiffman et al., 2019). The 50% retention threshold was selected as a pragmatic dimensionality-reduction criterion consistent with existing metabolomics filtering practices. Importantly, this step did not use genotype, infection status, statistical significance, or class separation. Among the remaining metabolites, the 50% with the highest variance were then retained. These metabolites accounted for 94% of the total variance in the intensity-filtered dataset (**Supplementary Figure 4**), resulting in a final dataset of 324 metabolites for bootstrap-enhanced LASSO analysis. The PCA was performed separately using the complete set of 1,294 QC-filtered metabolites.

### 2.7 Principal component analysis

Principal component analysis (PCA) was performed on the log-transformed and standardized dataset of 1,294 metabolites to assess the global structure of the metabolome using scikit-learn version 1.6.1 (Pedregosa et al., 2011). The percentage of variance explained by each principal component was extracted from the PCA model.

### 2.8 Bootstrap-enhanced LASSO

Discriminant metabolites (DMs) were identified by logistic regression with L1 penalty (LASSO) implemented in scikit-learn (version 1.6.1; Pedregosa et al., 2011) in Python (version 3.13.5). Model hyperparameters were tuned using six-fold cross-validation based on accuracy on the main dataset. In addition, cross-validation was also used to evaluate the internal classification performance of the model. To assess the stability of metabolite selection under sample resampling, a bootstrap procedure comprising 1,000 iterations was applied independently to each pairwise comparison. At each iteration, samples were resampled with replacement. The LASSO model with previously identified hyperparameters was fitted to the resampled dataset and metabolites presenting a non-zero coefficient were recorded. For each metabolite, the total selection frequency was calculated as the proportion of bootstrap iterations in which the metabolite was selected. Metabolites with a selection frequency > 30% were retained for the main analysis. This inclusive, dataset-specific threshold was retained based on the sensitivity analysis reported in **Supplementary Table 4**, which showed that more stringent cutoffs of > 40% and > 50% progressively reduced the number of retained metabolites and resulted in an overly sparse candidate set in some comparisons. The results obtained with these more stringent thresholds are reported in **Supplementary Table 4**. Following bootstrap-enhanced LASSO selection, metabolites corresponding to synthetic compounds or assigned to annotation confidence Levels 4–5 were excluded from downstream biological interpretation and pathway-level analyses. Only metabolites assigned to annotation confidence Levels 1–3 were retained as final DMs (**Supplementary Table 6**).

### 2.9 Model validation

The optimized model was fitted to the complete internal dataset and evaluated using an independent external dataset consisting of 12 samples (3 per class) extracted and processed in a similar way to the main dataset. Model performance metrics included precision, recall, F1-score, accuracy, and area under curve (AUC). Bootstrap-enhanced LASSO was performed independently for the following four comparisons: healthy lemon vs healthy orange, infected lemon vs infected orange, infected orange vs healthy orange, and infected lemon vs healthy lemon. Metabolites identified as discriminant were assigned to classes according to their mean regression coefficient calculated across bootstrap iterations.

### 2.10 Biosynthetic pathway mapping

To evaluate the metabolic relationships at the pathway level, DMs were projected onto phenylpropanoid and limonoid biosynthetic maps, based on KEGG map00940 for phenylpropanoids and map00941 for flavonoids (Kanehisa and Goto, 2000) and literature (Roy and Saraf, 2006).

### 2.11 Differentially abundant metabolites

Relative abundance of metabolites between different conditions was compared using Student’s *t*-test. The *p*-value was adjusted for multiple comparisons with Benjamini-Hochberg method (false discovery rate). Metabolites with a fold change (FC) ≥ 1.2 or FC ≤ 0.83 and adjusted *p*-value < 0.05 between comparisons were considered differentially accumulated.

### 2.12 Permutational multivariate analysis of variance

To evaluate the effects of genotype and infection on metabolome structure, permutational multivariate analysis of variance (PERMANOVA) was performed. Euclidean distance matrices were calculated from log2-transformed and standardized data. Permutational analysis of multivariate dispersions (PERMDISP) was performed to verify the homogeneity of the multivariate dispersion between groups. The significance of both tests was evaluated using 9,999 permutations. PERMANOVA and PERMDISP were performed using the ‘vegan’ package (version 2.7-3) in R (version 4.5.1) (Oksanen et al., 2026). Differences in the percentage of symptomatic leaves and *C*Las titers between infected lemon and infected sweet orange plants were assessed using two-sided Wilcoxon rank-sum tests. Effect sizes were calculated as $r=\frac{Z}{\surd N}$ where *Z* is the standardized Wilcoxon test statistic and *N* is the total number of observations.

## 3 Results

### 3.1 Phenotyping differences reveal differential susceptibility levels between lemon and orange

**Figure 1:**
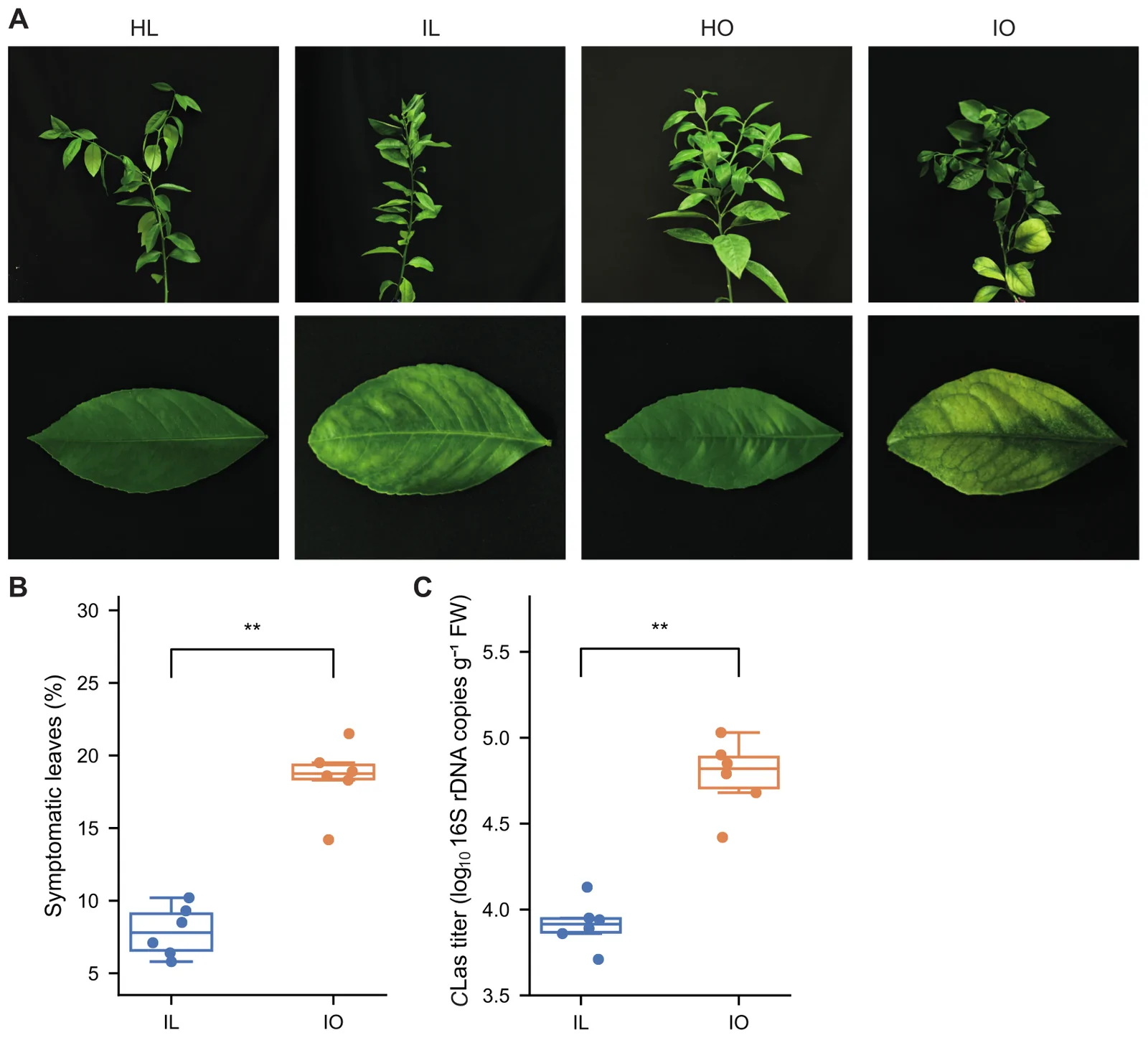
Phenotypic and pathogen load differences between lemon and orange. One-year-old lemon and orange plants were inoculated with *C*Las and analyzed at 24 weeks post-inoculation. **(A)** Whole plants and representative leaves from healthy and *C*Las-inoculated plants. **(B)** Frequency of symptomatic leaves in *C*Las-inoculated plants. **(C)** Bacterial load measured by quantitative PCR targeting the *C*Las 16S rDNA. Statistical significance was assessed using a Wilcoxon rank-sum test (**p < 0.01, n = 6 trees per group). HL, healthy lemon; IL, infected lemon; HO, healthy orange; IO, infected orange; FW, fresh weight.

Clear phenotypic differences were observed between *C*Las-inoculated lemon and orange plants at 24 weeks post-inoculation (wpi). While lemon plants were less severely affected, *C*Las-inoculated orange plants exhibited more pronounced disease symptoms, including reduced vigor and leaf chlorosis (**Figure 1A**). Lemon leaves only exhibited mild alterations, while orange leaves showed typical HLB symptoms characterized by interveinal chlorosis and blotchy mottling. In addition, the frequency of symptomatic leaves was higher in orange than in lemon plants (18% vs 7.5%) at 24 wpi (**Figure 1B**; **Supplementary Table 1**; Wilcoxon rank-sum test, *p* < 0.01, *r* = 0.838). These observations were consistent with qPCR results targeting the *C*Las 16S rDNA gene, which revealed a significantly lower pathogen load in lemon (3.91 ± 0.13 log_10_ copies g^-1^ FW) than in orange (4.77 ± 0.21 log_10_ copies g^-1^ FW; **Figure 1C**; **Supplementary Table 1**; Wilcoxon rank-sum test, *p* < 0.01, *r* = 0.832). Together, these results indicate that both varieties were successfully infected and orange plants exhibited more severe symptoms than lemon plants.

### 3.2 The global metabolome is primarily structured by genotype

**Figure 2:**
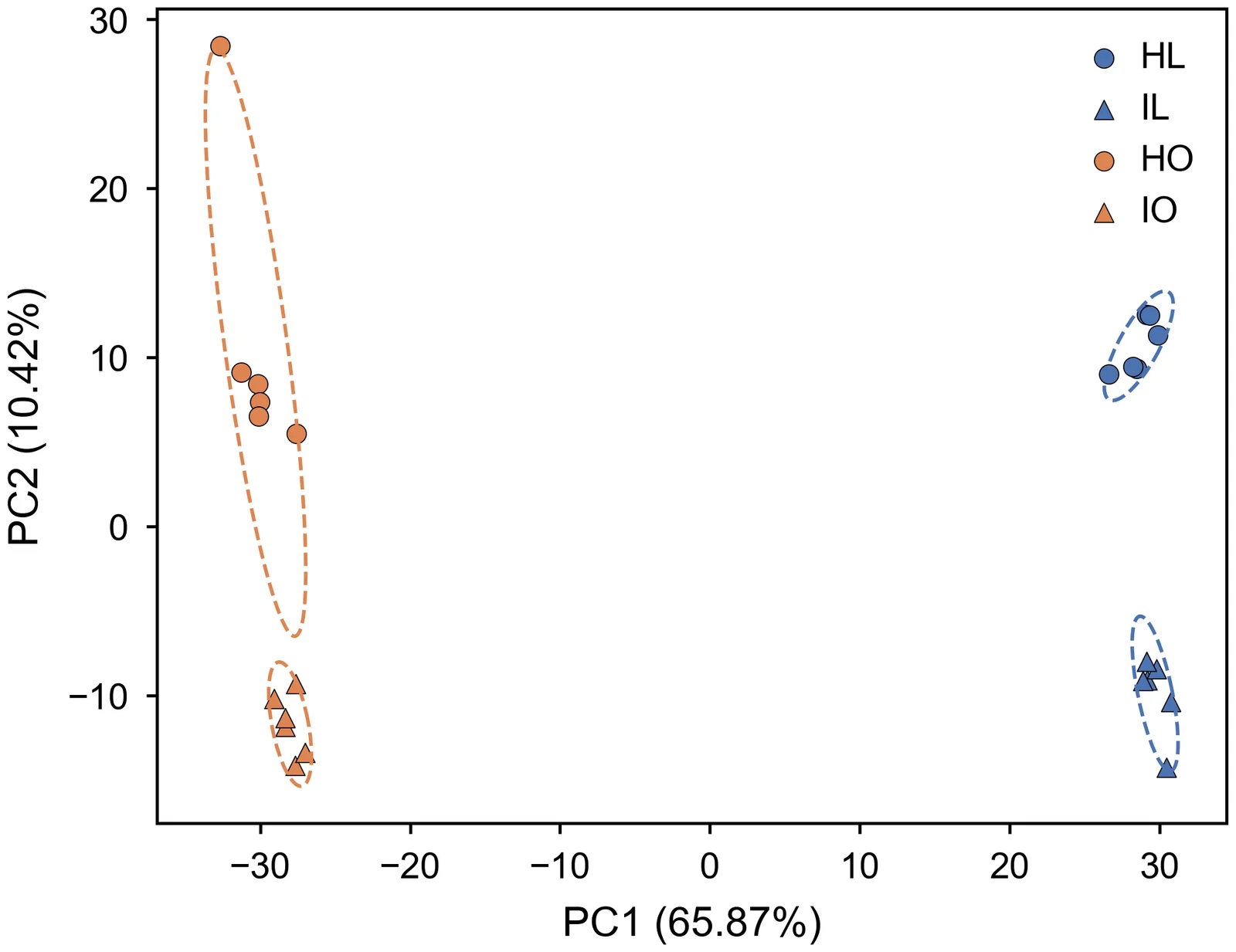
Global leaf metabolome structure in lemon and orange under healthy and infected conditions. Principal component analysis was performed on the 1,294 annotated metabolites detected by LC-MS in positive and negative ionization modes from leaves of lemon and orange under healthy conditions or at 24 weeks post-inoculation with *C*Las. Each point represents an individual biological replicate. Ellipses represent the dispersion of samples within each group (95% confidence; 2 standard deviations). HL, healthy lemon; IL, infected lemon; HO, healthy orange; IO, infected orange.

To assess the global structure of the metabolome in lemon and orange under healthy and *C*Las-infected conditions, we performed untargeted metabolomics using LC-MS in both positive and negative ion modes on leaf samples from healthy or *C*Las-infected plants. A total of 1,294 metabolites were identified following this method (**Supplementary Table 2**). A PCA was then performed on these 1,294 metabolites (**Figure 2**). The analysis included samples from four groups: healthy lemon, infected lemon, healthy orange, and infected orange. Samples formed distinct clusters for each group. A total of 76.29% of the variance was explained by the first two PCs, with PC1 explaining 65.87% and primarily separating samples according to their genotype. PC2 explained 10.42% of the variance and discriminated samples based on their infection status. The stronger effect of genotype was confirmed by PERMANOVA, which explained a large portion of the variance (*R^2^* = 0.658, *p* < 0.001; **Supplementary Table 3**), whereas the magnitude of infection-associated metabolic changes was lower (*R^2^* = 0.097, *p* < 0.001).

### 3.3 Bootstrap-enhanced LASSO identifies stable discriminant metabolites between orange and lemon

**Figure 3:**
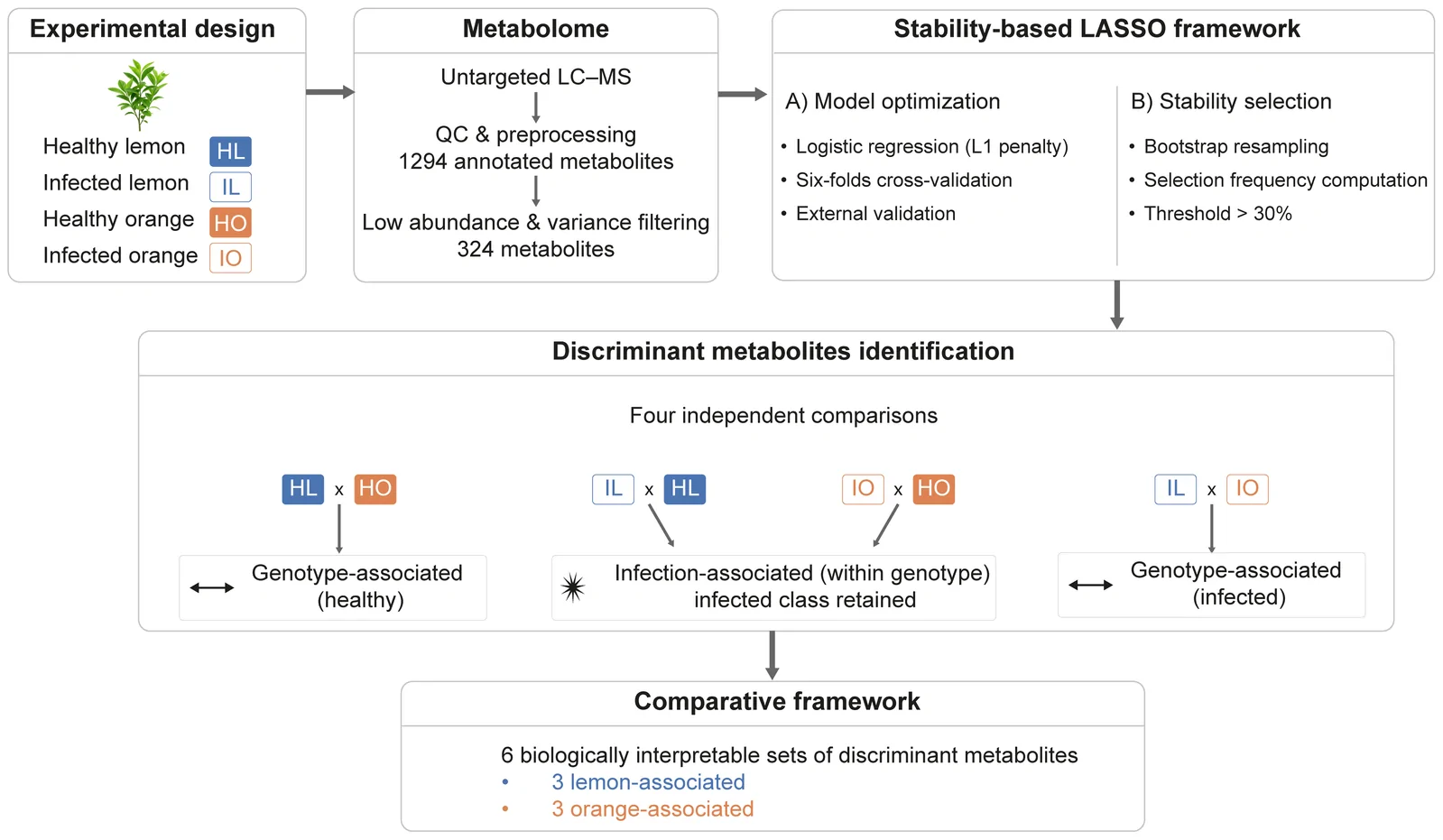
Experimental design and bootstrap-enhanced LASSO workflow used for the identification of discriminant metabolites. HL, healthy lemon; IL, infected lemon; HO, healthy orange; IO, infected orange.

To identify stable discriminant metabolites, we performed variable selection using logistic regression with L1 penalty (LASSO) combined with 1,000 bootstrap iterations, referred to as bootstrap-enhanced LASSO (**Figure 3**). We used a filtered dataset consisting of 324 metabolites, obtained after a preliminary feature filtering based on abundance and variance (see Materials and Methods). The variables selected during each bootstrap iteration were recorded, and metabolites were associated with a selection frequency across the bootstrap resamples. The selection frequency reflected the stability of variables during the resampling process and allowed the identification of the most consistent metabolites. Metabolites with a total selection frequency > 30% were retained as DMs for the main analysis. This threshold was used as an inclusive stability criterion to capture recurrently selected metabolites while avoiding excessive sparsity in a high-dimensional metabolomic dataset containing correlated features. To evaluate the influence of this selection-frequency cutoff, we performed a sensitivity analysis using increasingly stringent thresholds (> 30%, > 40% and > 50%; **Supplementary Table 4**). Increasing the threshold progressively reduced the number of retained DMs, with a stronger reduction in inter-genotype comparisons than in infection-associated comparisons. In particular, the healthy lemon vs healthy orange comparison retained no DMs above the > 50% threshold. These results supported the use of the > 30% threshold as a balanced cutoff for the main analysis, particularly for inter-genotype comparisons where more stringent thresholds led to excessive sparsity. Six-fold cross-validation was used to optimize model hyperparameters and evaluate internal classification performance. All models exhibited an AUC of 1.00 during cross-validation (**Supplementary Figure 1**). In addition, the ability of the model to generalize was evaluated using an independent external dataset. Classification accuracy ranged from 83.33% to 100%, with AUC values ranging from 0.89 for intra-genotype comparisons to 1.00 for inter-genotype comparisons (**Supplementary Figure 2**; **Supplementary Table 5**).

**Figure 4:**
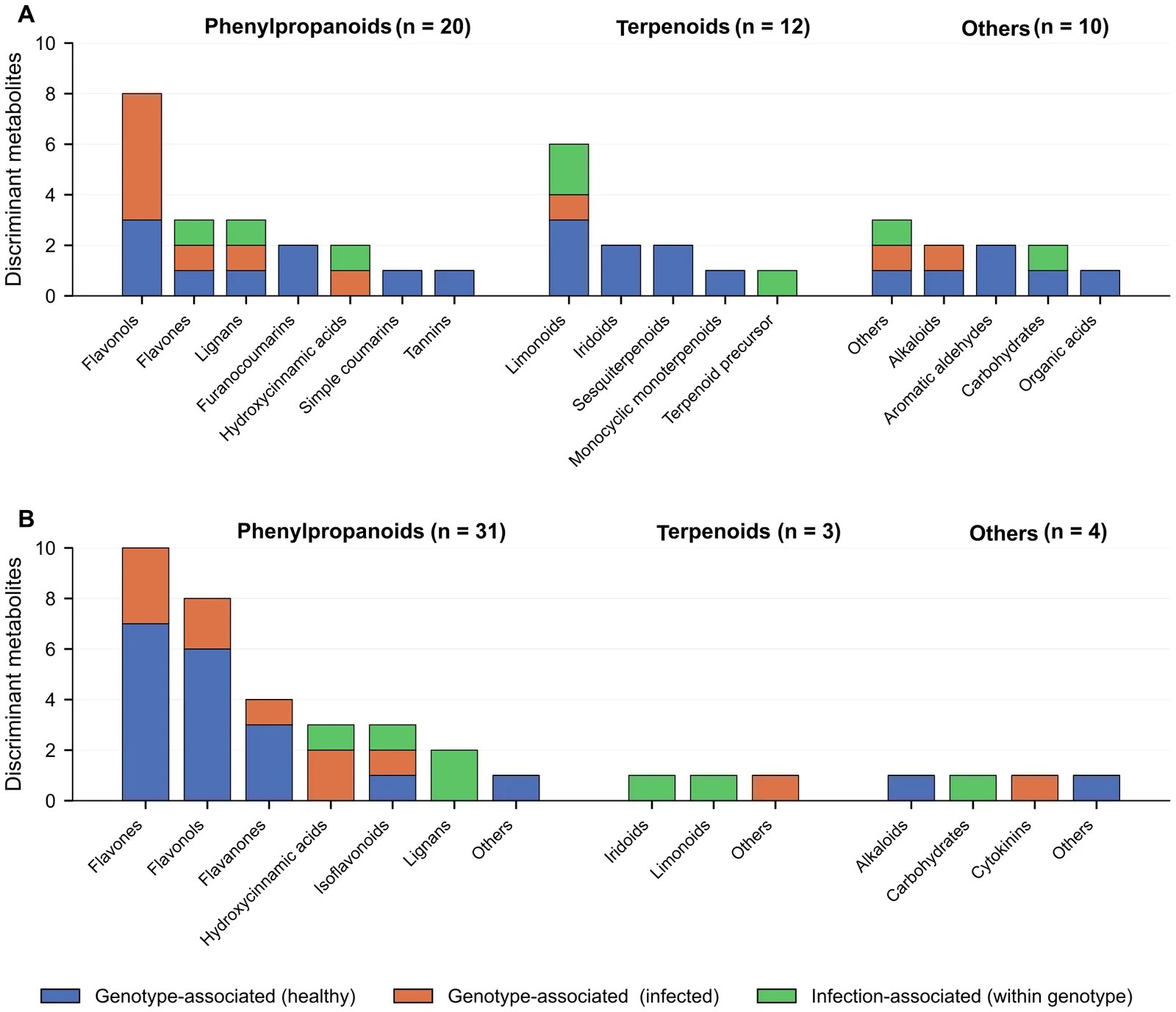
Distribution of discriminant metabolites (DMs) in lemon and orange. DMs associated with **(A)** lemon and **(B)** orange are grouped according to three comparative frameworks: genotype-associated (healthy), genotype-associated (infected) and infection-associated (within genotype). DMs are organized into major metabolic classes (phenylpropanoids, terpenoids, and other classes) and further subdivided into specific subclasses.

Bootstrap-enhanced LASSO was independently applied to four comparisons: healthy lemon vs healthy orange, infected lemon vs infected orange, infected lemon vs healthy lemon, and infected orange vs healthy orange. A total of 79, 48, 46, and 36 metabolites were identified, respectively. Among them, metabolites of synthetic origin or with insufficient annotation confidence for biological interpretation were excluded from subsequent analysis. This additional filtering step retained 42, 23, 24, and 15 metabolites, respectively, which were considered as DMs. Within each of these comparisons, the identified DMs were assigned to one of the two classes based on the sign of their mean LASSO regression coefficient calculated across bootstrap iterations (**Supplementary Table 6**). The distribution of DMs across metabolic classes for lemon and orange is shown in **Figures 4A, B**, respectively. The comparison between healthy lemon and healthy orange provided information about the constitutive differences between both varieties under healthy conditions. A total of 42 DMs were identified, of which 23 were positively associated with healthy lemon and 19 associated with healthy orange. Lemon-associated metabolites mainly belonged to phenylpropanoids and terpenoids. Phenylpropanoids were predominantly represented by flavonoids, particularly flavonols, and by coumarins, mainly furanocoumarins. Among terpenoids, triterpenoid limonoids were the most represented. In contrast, metabolites associated with healthy orange were almost exclusively phenylpropanoids, primarily flavonoids distributed across several subclasses, such as flavonols, flavanones, and flavones. Next, the comparison between infected lemon and infected orange revealed genotype-associated differences during the infection. Twenty-three DMs were identified, of which 11 were associated with infected lemon and 12 with infected orange. In lemon, DMs were mainly flavonoids, more precisely flavonols, together with one hydroxycinnamic acid (HCA). A similar predominance of flavonoids was observed with infected orange. Finally, to gain insight into the metabolic changes associated with infection within each genotype, we compared infected lemon with healthy lemon and infected orange with healthy orange. Only metabolites associated with the infected classes were retained for further analysis. In lemon, 24 DMs were identified, of which 8 were associated with infection, including 2 limonoids. In orange, 15 DMs were identified, and 7 were associated with infection, including 4 phenylpropanoids and 1 limonoid. In total, these four comparisons provided a total of six sets of DMs, three being associated with lemon and three with orange, and each of them providing distinct information.

### 3.4 Contrasting organization of phenylpropanoid metabolism between lemon and sweet orange

**Figure 5:**
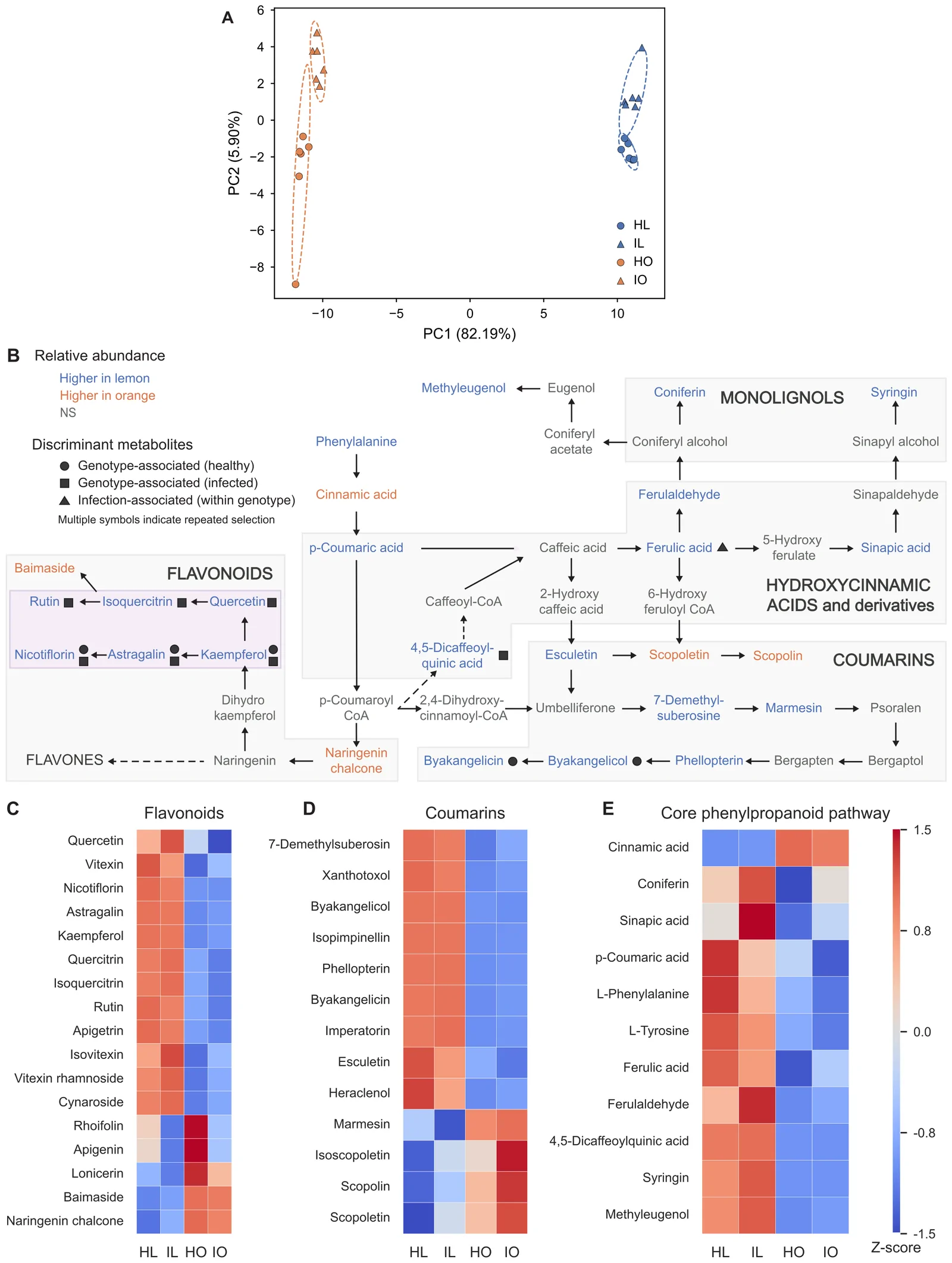
Phenylpropanoid metabolic profiles in lemon and orange under healthy and infected conditions. **(A)** Phenylpropanoid biosynthetic pathway (adapted from KEGG map00940 and map00941). Text color indicates differentially abundant metabolites between lemon and orange. Symbols show discriminant metabolites identified across the three comparative frameworks. The quercetin and kaempferol branches were highlighted with a light pink shaded box to improve readability. **(B-D)** Heatmaps showing normalized relative abundance (z-score) of metabolites belonging to **(B)** flavonoids, **(C)** coumarins, and **(D)** core phenylpropanoid pathway in four sample groups. NS, not significant; HL, healthy lemon; IL, infected lemon; HO, healthy orange; IO, infected orange.

Among the different metabolite classes identified within DMs, phenylpropanoids emerged as the most represented across genotypes and infection conditions (**Figures 4A, B**). This observation led us to further investigate this broad class of metabolites. The phenylpropanoid pathway comprises several subclasses of secondary metabolites, including flavonoids, coumarins, and HCAs, which are well known for their involvement in biotic stress responses (Dixon et al., 2002; Hijaz et al., 2020). To determine if DMs were randomly distributed or organized within specific metabolic branches, we constructed a phenylpropanoid biosynthetic map including multiple subclasses. Within flavonoids, DMs were predominantly enriched in flavonols (**Figure 5A**). Flavonols were generally more abundant in lemon than in orange, in both healthy and infected samples, highlighting constitutive differences between both genotypes (**Figure 5B**). Additionally, their overall accumulation remained stable after infection in lemon. Notably, all the flavonols identified as DMs in lemon were localized within similar biosynthetic branches centered around the aglycones kaempferol and quercetin, as well as their glycosylated products, including astragalin, nicotiflorin, isoquercitrin, and rutin (**Figure 5A**). The kaempferol branch was also identified as discriminant for lemon in both inter-genotype comparisons (healthy and infected). Conversely, orange-associated flavonol DMs were distributed across several aglycone backbones, indicating a more dispersed organization than the branch-focused pattern observed in lemon (**Supplementary Table 6**). This dispersed organization was maintained under a less stringent LASSO-based selection (threshold = 20%), supporting that the orange-associated flavonol profile was not solely an artifact of the 30% selection threshold (**Supplementary Table 7**). In addition, several orange-associated flavonol DMs were derived from methylated aglycones, whereas no methylated-aglycone-derived flavonol was detected among lemon-associated flavonol DMs. The abundance of the quercetin branch (including isoquercitrin and rutin) was decreased upon infection in orange, indicating that *C*Las modulates specific flavonol branches in orange (**Figure 5B**).

Within coumarins, furanocoumarins were consistently more abundant in lemon than in orange, in both healthy and infected plants (**Figure 5C**). Infection-related changes were limited, indicating that the main difference arose from their constitutive levels between genotypes. Byakangelicin and its precursor byakangelicol, two linear furanocoumarins, were identified as lemon-associated DMs in the comparison between healthy lemon and healthy orange (**Figure 5A**). The identification of a precursor-product pair as DMs between healthy lemon and healthy orange supports the association of this specific furanocoumarin branch with the lemon metabolic profile.

Among HCAs and derivatives, four out of six members showed significant changes upon infection (**Figure 5D**). Within this group, p-coumaric acid and ferulic acid exhibited contrasting profiles. The accumulation of p-coumaric acid was reduced in both lemon and orange after infection. In contrast, ferulic acid levels were decreased in lemon but increased in orange upon infection, while maintaining an overall higher accumulation in lemon. Notably, ferulic acid was identified as an infection-associated DM in orange (infected orange vs healthy orange) but its accumulation remained higher in lemon during infection (**Figure 5A**). Although HCAs exhibited a more pronounced response to infection compared with flavonols or furanocoumarins, they still maintained a stable inter-genotype differential abundance. Ferulic acid and, more generally, HCAs are key intermediates in cell wall reinforcement processes and serve as precursors for monolignol biosynthesis (Reem et al., 2016; Vanholme et al., 2010). Although no monolignols were identified as DMs, we investigated downstream HCA-derived metabolites. Coniferin and syringin, two monolignol glycosides, showed a higher accumulation in lemon than in orange in both healthy and infected samples (**Figure 5D**). Upon infection, their abundance was further increased in lemon, whereas only coniferin increased in orange.

Overall, the phenylpropanoid metabolism appeared as primarily structured by genotype, with several subclasses exhibiting stable constitutive differences between lemon and orange. While infection induced quantitative changes, the relative inter-genotype differential abundance remained preserved, suggesting the infection-induced modulation operates within a pre-organized metabolic architecture.

### 3.5 Organization of limonoid metabolism: constitutive enrichment and infection-related modulation

**Figure 6:**
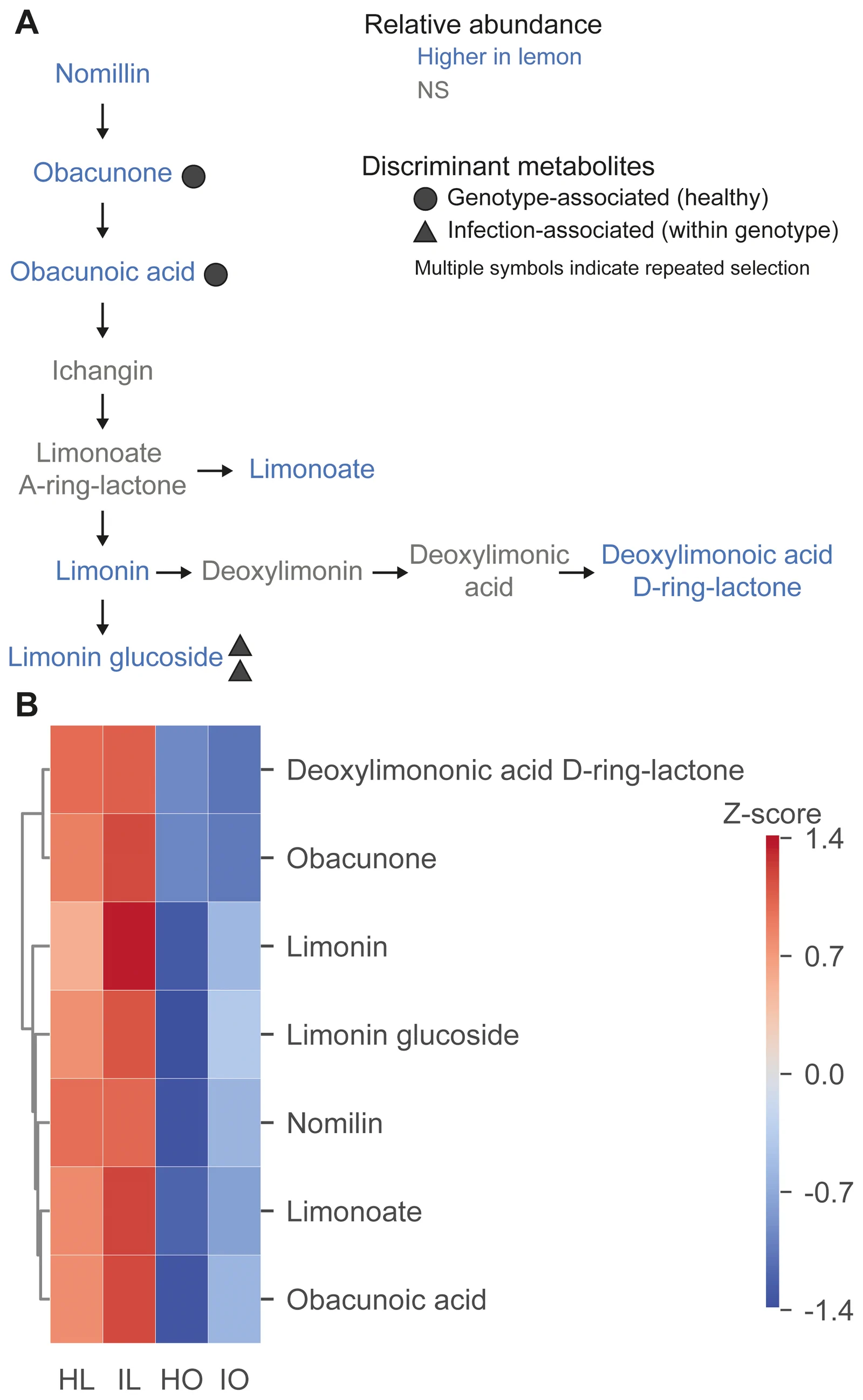
Limonoid metabolic profiles in lemon and orange under healthy and infected conditions. **(A)** Schematic representation of the limonoid biosynthetic pathway, adapted from (Roy and Saraf, 2006). Text color indicates differentially abundant metabolites between lemon and orange. Symbols indicate discriminant metabolites identified across the three comparative frameworks. **(B)** Heatmap showing the normalized relative abundance (z-score) of limonoids. NS, not significant; HL, healthy lemon; IL, infected lemon; HO, healthy orange; IO, infected orange.

While phenylpropanoids formed an important discriminant backbone between genotypes, limonoids also emerged from the DMs. Limonoids are highly oxygenated tetranortriterpenoids, particularly abundant in *Citrus* spp. (Manners, 2007). and associated with defense-related functions (Vikram et al., 2010; Ruberto et al., 2002). Multiple limonoids were identified as DMs (**Figures 4A, B**) and mapped onto a limonoid biosynthetic map (Roy and Saraf, 2006) in order to assess their biosynthetic relationships and pathway-level distribution. Overall, lemon samples exhibited a higher accumulation of limonoids compared with orange, whether infected or not (**Figure 6A**). In addition, limonoid accumulation was significantly increased upon infection in both genotypes (**Figure 6B**; **Supplementary Figure 3**; **Supplementary Table 8**), whereas no significant genotype × infection interaction was observed (PERMANOVA, *p* = 0.12), indicating that changes in limonoid accumulation upon infection were similar in both varieties. In the comparison between healthy lemon and healthy orange, three limonoids were identified as lemon-associated DMs, including obacunone and obacunoic acid, two biosynthetically related metabolites (**Figure 6A**). Additionally, two infection-associated limonoid DMs were identified in the comparison between infected lemon and healthy lemon, including limonin-glucoside. Limonin-glucoside is biosynthetically related to obacunone and obacunoic acid, identified as constitutive DMs, indicating that infection-associated variations occur within the same limonoid branch. Notably, limonin-glucoside was also found as a marker of infection in orange in the comparison between infected orange and healthy orange. Together, these observations suggest that limonoid metabolism is characterized by constitutive differences between genotypes and a significant infection-associated modulation in both genotypes, with an overall higher accumulation in lemon.

## 4 Discussion

### 4.1 Distinct metabolic organization between lemon and orange is associated with contrasting HLB responses

HLB is a phloem-limited disease that induces profound physiological and metabolic perturbations in citrus. While several studies have described metabolomic changes associated with infection (Li et al., 2024), the relative contribution of the constitutive metabolic architecture compared with infection-induced changes in the context of HLB tolerance remains unclear. In this study, comparative metabolomic analyses showed that tolerant lemon and susceptible orange exhibited markedly distinct metabolic organizations. Lemon displayed higher basal levels of flavonols, furanocoumarins, limonoids, and metabolites related to cell wall reinforcement compared with orange. Although *C*Las infection modulated some specific branches, it did not alter the major metabolic differences observed between the two cultivars. The existence of constitutive differences between genotypes, combined with a coherent organization of the DMs within phenylpropanoid and limonoid branches, suggests that the milder HLB phenotype observed in lemon could be associated with a pre-existing metabolic architecture, rather than solely with infection-induced responses.

### 4.2 A structured flavonol module associated with the lemon genotype

Among the total pool of DMs, flavonols emerged as highly represented (**Figures 4A, B**). In the comparison between healthy lemon and healthy orange, lemon-associated flavonols were clustered around the kaempferol branch (kaempferol, astragalin, and nicotiflorin; **Figure 5A**). However, orange-associated flavonols were distributed across several aglycone backbones, indicating a more structured organization in lemon and a more dispersed profile in orange (**Supplementary Table 6**). This dispersed organization was maintained under a relaxed selection-frequency threshold (> 20%), suggesting that it was not solely an artifact of the LASSO cutoff (**Supplementary Table 7**). Notably, several orange-associated flavonols were derived from methylated aglycones, including limocitrol, larycitrin, isorhamnetin, and rhamnocitrin, whereas lemon-associated flavonols were restricted to kaempferol- and quercetin-derived metabolites. Methylation of flavonol hydroxyl groups was shown to attenuate antioxidant capacity by reducing deprotonation-dependent electron donation, thereby lowering radical-scavenging efficiency (Lemańska et al., 2004). Therefore, the orange-associated flavonol profile may differ from the lemon-associated kaempferol/quercetin module not only in branch organization, but also in potential redox functionality.

In the comparison between infected lemon and infected orange, the kaempferol branch remained discriminant, while most flavonols previously associated with healthy orange were no longer detected as DMs (**Supplementary Table 6**). This observation suggests a more stable lemon-associated flavonol signature upon infection, whereas the orange-associated flavonol profile appeared less conserved under infected conditions. Alongside the kaempferol branch, the quercetin branch (isoquercitrin and rutin) emerged as discriminant in the comparison between infected lemon and infected orange. A closer examination of the abundance patterns revealed that both kaempferol and quercetin branches were more abundant in lemon regardless of infection status, and that the abundance remained stable upon infection (**Figure 5B**). In contrast, the levels of the quercetin branch were decreased in orange during infection. The stability and persistence of these two branches raise the question of their potential function in the context of HLB tolerance. Flavonols are well known for their antioxidant properties, with the ability to regulate redox homeostasis and reduce the accumulation of ROS (Agati et al., 2012). Notably, rutin, identified as a DM within the quercetin branch, has previously been shown to reduce HLB symptoms by reducing H_2_O_2_ accumulation when applied by foliar spray in infected orange trees, highlighting the biological coherence of this branch (Ma et al., 2022). In addition, quercetin derivatives have been reported to exhibit antibacterial activities, by directly binding the essential ribonuclease YbeY of *C*Las and inhibiting its activity, thereby impairing its rRNA maturation and ribosome biogenesis in infected tissues (Zuo et al., 2019). These findings highlight this module as a relevant target for further investigation in the HLB pathosystem. Together, these results identify a structured kaempferol/quercetin module characterized by higher and more stable abundance in lemon than in orange.

However, ROS accumulation, oxidative damage, and direct antibacterial activity were not measured in the present study. Therefore, the proposed roles of the kaempferol/quercetin module in redox regulation or bacterial inhibition should be considered as functional hypotheses supported by metabolite identity and previous literature, rather than as directly demonstrated mechanisms.

### 4.3 Structural defenses: cell wall reinforcement and phenylpropanoids

HCAs represent a versatile subclass of phenylpropanoids involved in antimicrobial responses, cell wall reinforcement and defense-related signaling, while also serving as precursors for other defense-related metabolites such as coumarins and monolignols (Vanholme et al., 2010; Von Roepenack-Lahaye et al., 2003; Kahramanoglu and Usanmaz, 2021). In our dataset, HCAs were generally more abundant in lemon than in orange, in both healthy and infected plants, revealing a constitutive enrichment of this metabolic branch in lemon (**Figure 5D**). These observations are consistent with previous reports highlighting that tolerant varieties naturally accumulate higher amounts of HCAs, especially ferulic, sinapic, and p-coumaric acids (Hijaz et al., 2020). The abundance profile of HCAs revealed a complex and dynamic regulation during HLB infection. Although sinapic acid and ferulaldehyde levels increased upon infection, p-coumaric acid levels decreased. Notably, ferulic acid showed reduced levels in infected lemon but increased in infected orange. In addition, ferulic acid was identified as an infection-associated DM in orange (**Figure 5A**). This opposite pattern observed for ferulic acid could reflect different utilization strategies for this key molecule between both varieties upon infection. One possible interpretation is that the decrease observed in lemon reflects increased utilization of its soluble ferulic acid pool, whereas orange accumulates ferulic acid after infection; however, metabolite fluxes were not measured in this study. Such a dynamic has been described in sugarcane, where cultivars resistant to *Sporisorium scitamineum* showed a quick and strong redistribution of their constitutive ferulic acid pool toward cell wall reinforcement after infection through polysaccharide cross-linking; in contrast, no significant changes were observed in susceptible cultivars (Santiago et al., 2009). The higher constitutive ferulic acid pool in lemon may represent a larger precursor pool. This interpretation is consistent with transcriptomic evidence showing that HLB-tolerant varieties exhibit a stronger and earlier activation of enzymes related to biosynthesis and modification of the cell wall (Gao et al., 2023). A larger constitutive ferulic acid pool may facilitate such a response. The decreased abundance of soluble ferulic acid after infection in lemon may also be consistent with its conversion into downstream or cell-wall-bound products. However, cell-wall-bound phenolics were not quantified, and this hypothesis cannot be resolved using the present solvent-based LC-MS dataset (Doppler et al., 2022). Additionally, the increased abundance of ferulaldehyde and sinapic acid after infection is compatible with altered HCA and monolignol-related metabolism, although it does not demonstrate increased lignin biosynthesis (**Figure 5D**).

Lignin is an essential phenolic polymer of the cell wall, playing a key role in the mechanical support of vascular tissues and providing resistance to pathogens (Vanholme et al., 2010). In our metabolome, coniferin and syringin, the glycosylated forms of the monolignols coniferyl alcohol and sinapyl alcohol, showed higher accumulation in lemon than in orange, in both healthy and infected plants (**Figure 5D**). In addition, their accumulation was increased upon infection in lemon, while only coniferin showed increased levels in orange. *C*Las infection is associated with a progressive degeneration of phloem sieve tubes, including sieve plate obstruction and the collapse of sieve elements and companion cells. This phenomenon is usually more pronounced in susceptible cultivars (Fan et al., 2013). Lignin was shown to play a key role in providing mechanical support to vascular tissues (Muro-Villanueva et al., 2019). While no direct evidence has been reported linking lignification and phloem stability in the context of HLB, increased lignification of vascular tissues has been associated with increased resistance to vascular pathogens in the tomato-*Ralstonia solanacearum* pathosystem (Shi et al., 2023). Monolignols are often stored in glycosylated form to reduce their phytotoxicity, while facilitating their transport and storage. This strategy allows rapid release of active monolignols by deglycosylation when defense responses are required (Baiya et al., 2021). The higher constitutive abundance of coniferin and syringin in lemon indicates a larger pool of monolignol glycosides. Whether this pool is mobilized during infection or contributes to cell wall reinforcement remains to be functionally determined.

### 4.4 Constitutive and infection-associated patterns of coumarins and limonoids

Coumarins are a major class of plant secondary metabolites produced from the phenylpropanoid pathway and are involved in defense mechanisms against a wide variety of pathogens such as bacteria, viruses, fungi, oomycetes, and insects (Stringlis et al., 2019). Among coumarins, furanocoumarins accumulated at higher levels in lemon, with infection showing no significant change in their accumulation (**Figure 5C**). A similar pattern was observed in orange, but at reduced levels. In particular, byakangelicol and byakangelicin, two biosynthetically related metabolites, were identified as lemon-associated DMs in the comparison between healthy lemon and healthy orange, indicating a significant constitutive difference in this furanocoumarin branch (**Figure 5A**). Lemon is known to accumulate higher levels of furanocoumarins (Dugrand-Judek et al., 2015). In particular, the linear cluster of furanocoumarins, which includes byakangelicol and byakangelicin, is known to be involved in both constitutive and induced plant defense, acting as either phytoanticipins or phytoalexins (Bruni et al., 2019). For example, 8-methoxypsoralen and isopimpinellin were shown to penetrate fungal conidia of *Colletotrichum acutatum* and induce oxidative damage upon UV exposure (De Menezes et al., 2014). Whether the higher constitutive abundance of these furanocoumarins in lemon has a functional role during CLas infection remains to be determined.

Limonoids are a subclass of highly oxygenated triterpenoids mainly occurring in the *Rutaceae* and *Meliaceae* families, with a particularly high abundance in Citrus species (Manners, 2007). *C. sinensis* phloem sap is known to be enriched in limonoids, particularly nomilin and limonin (Hijaz et al., 2016). In our study, limonoids were generally more abundant in lemon than in orange, in both infected and healthy tissues (**Figure 6B**). Unlike the metabolite classes discussed above, overall limonoid abundance increased significantly after *C*Las infection in both genotypes. Obacunone and obacunoic acid, two biosynthetically related limonoids, were associated with lemon in the healthy lemon vs healthy orange comparison (**Figure 6A**). Conversely, limonin-glucoside emerged as an infection-associated metabolite in both genotypes. Together, these observations show that limonoid abundance increased after infection in both genotypes and remained higher overall in lemon. Whether this metabolic pattern contributes functionally to the contrasting HLB phenotypes remains to be established. This pattern combines constitutively higher limonoid abundance in lemon with infection-associated increases in both genotypes. Limonoids are mainly known for their anti-insect activity (Ruberto et al., 2002), but some studies also suggest that specific limonoids can affect bacterial behavior. For example, obacunone was reported to interfere with quorum-sensing-regulated processes and biofilm accumulation in *Vibrio harveyi* (Vikram et al., 2010). However, whether similar effects occur during *C*Las infection remains unknown. Limonoid accumulation in fruits has been extensively studied, as they are the source of the bitter taste characterizing infected fruits (Roy and Saraf, 2006; Gaikwad et al., 2025). However, limonoid accumulation patterns in other tissues are not well documented. In addition, the potential link between limonoids and defense against HLB has received little focus. Multiple metabolomic studies have compared susceptible and tolerant citrus varieties upon *C*Las infection, but little focus has been placed on limonoids despite being reported as more abundant in tolerant cultivars (Mahmoud et al., 2023). Our results extend the characterization of limonoid metabolism beyond fruits, revealing both constitutive differences between citrus genotypes and a shared infection-associated response in leaves.

### 4.5 Methodological robustness and limitations

Metabolomic studies often involve datasets where the number of metabolites (p) is considerably higher than the number of samples (n). In such situations, models based on PLS regression are efficient to explore the global structure of the dataset, but are prone to overfitting and offer limited stability for the identification of individual biomarkers (Worley and Powers, 2013). In contrast, penalized models such as LASSO perform an explicit variable selection, facilitating interpretability and biomarker identification between classes (Tibshirani, 1996). However, when p >> n and predictors are highly correlated, LASSO itself can still provide unstable results (Meinshausen and Bühlmann, 2010). In this work, we coupled LASSO with a bootstrap strategy to select variables based on their frequency of selection across 1,000 resampled models, thereby prioritizing stable metabolites and reducing the risk of false positives (**Figure 3**). Given the limited sample size, the high classification performance should be interpreted as reflecting strong separation within the present dataset rather than as definitive evidence of broad predictive generalizability. The selected DMs were therefore treated as an initial exploratory candidate set and further evaluated based on their abundance patterns, biosynthetic coherence, and consistency with previous biological evidence. As a result, the DMs identified with this method often converged into biologically meaningful biosynthetic branches, such as the kaempferol and quercetin branches, the linear furanocoumarins, and limonoids. While individual metabolite selection may be influenced by the correlation structures inherent to high-dimensional datasets, the consistent identification of metabolic branches across comparisons strengthens the robustness of the underlying biological pattern.

Evaluating *C*Las infection at 24 wpi captured a stage characterized by a sustained defense-related reprogramming, notably involving the phenylpropanoid pathway (Tian et al., 2026). While this timing is biologically relevant, it is important to note that it may miss some transient early responses. For example, some flavonoids were shown to accumulate in early stages of infection (Li et al., 2024). In addition, an early activation of enzymes related to biosynthesis and modification of the cell wall was observed in HLB-tolerant varieties (Gao et al., 2023).

An important limitation is that the present study compares only one tolerant lemon cultivar with one susceptible sweet orange cultivar. Therefore, the genotype-dependent metabolic patterns identified here cannot yet be generalized to citrus tolerance more broadly and should be validated across a wider panel of tolerant and susceptible genotypes. In addition, although the candidate metabolic features were supported by consistent abundance patterns, stable selection through bootstrap-enhanced LASSO, and pathway-level coherence, their functional relevance to HLB tolerance remains to be established experimentally.

### 4.6 Implications and future perspectives

Taken together, our results show that the contrasting HLB responses of lemon and orange are associated not only with infection-induced metabolic changes, but also with marked constitutive differences in metabolic organization. In this two-genotype comparison, lemon exhibited a pre-existing enrichment in several defense-related metabolic branches, including flavonols, furanocoumarins, monolignol-related metabolites, and limonoids. These findings suggest that constitutive metabolic profiles may represent useful traits to explore in future studies of HLB tolerance. However, broader screening across citrus germplasm and deeper functional validation will be required to determine whether these metabolic features are causally involved in HLB tolerance and whether they can be used in selection or breeding strategies.

## Data Availability

The dataset used in this study is provided in **Supplementary Table 2**. The code used for the machine-learning analysis is publicly available on Figshare at https://doi.org/10.6084/m9.figshare.31587988.
