# Supplementary material for: Distinct metabolic architectures differentiate HLB-tolerant lemon and susceptible sweet orange
Source: Front Plant Sci. 2026 Aug 11;17:1905341. doi: 10.3389/fpls.2026.1905341 (PMC13503365; doi:10.3389/fpls.2026.1905341)
Supplement: Supplementary file 1 [file DataSheet1.docx]

**Figure S1.** ROC curves and AUC values obtained using 6-fold cross-validation.

**Figure S2.** ROC curves and AUC values obtained using an independent external dataset.

**Figure S3.** Accumulation of limonoids across the four sample classes.

**Figure S4.** Cumulative variance explained by metabolites ranked according to variance across samples.

**Figure S1.** ROC curves and AUC values obtained using 6-fold cross-validation. (A) healthy lemon vs healthy orange, (B) infected lemon vs healthy lemon, (C) infected lemon vs infected orange, and (D) infected orange vs healthy orange.

**Figure S2**. ROC curves and AUC values obtained using an independent external dataset. (A) healthy lemon vs healthy orange, (B) infected lemon vs healthy lemon, (C) infected lemon vs infected orange, and (D) infected orange vs healthy orange.

**Figure S3.** Accumulation of limonoids across the four sample classes. Each dot represents an individual limonoid. Asterisks indicate significant pairwise differences in limonoid profiles based on PERMANOVA comparisons (*p* < 0.01; n = 6 biological replicates per group; Supplementary Table S8). Horizontal bars represent the median abundance of limonoids within each group. HL, healthy lemon; IL, infected lemon; HO, healthy orange; IO, infected orange.

**Figure S4.** Cumulative variance explained by metabolites ranked according to variance across samples. The 50% most variable metabolites account for 94% of the total variance.
